# Biocompatible micro tweezers for 3D hydrogel organoid array mechanical characterization

**DOI:** 10.1371/journal.pone.0262950

**Published:** 2022-01-24

**Authors:** Soliman Alhudaithy, Kazunori Hoshino

**Affiliations:** 1 Department of Biomedical Engineering, University of Connecticut, Storrs, Connecticut, United States of America; 2 Department of Biomedical Technology, King Saud University, Riyadh, KSA; University of South Carolina, UNITED STATES

## Abstract

This study presents novel biocompatible Polydimethylsiloxane (PDMS)-based micromechanical tweezers (μTweezers) capable of the stiffness characterization and manipulation of hydrogel-based organoids. The system showed great potential for complementing established mechanical characterization methods such as Atomic Force Microscopy (AFM), parallel plate compression (PPC), and nanoindentation, while significantly reducing the volume of valuable hydrogels used for testing. We achieved a volume reduction of ~0.22 μl/sample using the μTweezers vs. ~157 μl/sample using the PPC, while targeting high-throughput measurement of widely adopted micro-mesoscale (a few hundred μm-1500 μm) 3D cell cultures. The μTweezers applied and measured nano-millinewton forces through cantilever’ deflection with high linearity and tunability for different applications; the assembly is compatible with typical inverted optical microscopes and fit on standard tissue culture Petri dishes, allowing mechanical compression characterization of arrayed 3D hydrogel-based organoids in a high throughput manner. The average achievable output per group was 40 tests per hour, where 20 organoids and 20 reference images in one 35 mm petri dish were tested, illustrating efficient productivity to match the increasing demand on 3D organoids’ applications. The changes in stiffness of collagen I hydrogel organoids in four conditions were measured, with ovarian cancer cells (SKOV3) or without (control). The Young’s modulus of the control group (Control—day 0, E = 407± 146, n = 4) measured by PPC was used as a reference modulus, where the relative elastic compressive modulus of the other groups based on the stiffness measurements was also calculated (control-day 0, E = 407 Pa), (SKOV3-day 0, E = 318 Pa), (control-day 5, E = 528 Pa), and (SKOV3-day 5, E = 376 Pa). The SKOV3-embedded hydrogel-based organoids had more shrinkage and lowered moduli on day 0 and day 5 than controls, consistently, while SKOV3 embedded organoids increased in stiffness in a similar trend to the collagen I control from day 0 to day 5. The proposed method can contribute to the biomedical, biochemical, and regenerative engineering fields, where bulk mechanical characterization is of interest. The μTweezers will also provide attractive design and application concepts to soft membrane-micro 3D robotics, sensors, and actuators.

## 1. Introduction

### 1.1. Background

The lack of cell-matrix interaction in typical 2D culture models compared to 3D models illustrates the importance of 3D biomimetic environment models [1, 2]. For instance, studies have reported increased drug resistance in 3D cultures compared to 2D monolayer models, indicating that 3D models better represent *in vivo* conditions [3, 4]. The use of 3D models has shown great potential in studying different types of cancer, developing a better understanding of the 3D environmental cellular cues and signals [2], supporting the existing therapeutic approaches, and creating novel targeted precision medicine approaches [5].

As one example, Epithelial Ovarian Cancer (EOC) is among the most lethal diseases for women. Most women are diagnosed with late-stage (III/IV) disease, and unfortunately, high percentages of late-stage diagnosed patients are in danger of dying of their disease. Early and rapid metastasis is considered one of the leading causes of high lethality. The tumor 3D organization and microenvironment peculiarities are linked with the tumor metastasis and resistance to therapy [4]. *In vitro* models that mimic such a 3D microenvironment is crucial to find better treatment. On the cellular level, the substrate or hydrogel matrix has a significant effect on the status of the cultured cells. Parameters that characterize extracellular matrix (ECM) include substrate structure, source, type [6], fiber mesh, density, porosity, diffusivity, attachment site characteristics, physical and chemical cross-linkers, incorporated growth factors, supplements, medium, and matrix stiffness [7]. Similarly, pH, ionic concentration, and temperature can influence ECM architecture and collagen polymerization. All together constitute a complex microenvironment that affects how the cultured cells respond [7, 8]. The cell phenotype, including geometry and morphology, were reported to have strong correlations to the microenvironment. Many have investigated substrate stiffness as the environmental parameters on the cell morphology (acini, rounded, protrusions, or invasive). Different epithelial cancer cell lines have shown various morphological cellular behaviors in response to substrate stiffness [9].

The matrix stiffness has been well reported to signal stem cell lineage and phenotype commitment with extreme sensitivity to tissue level elasticity [10]. Mechanical characterization of the substrate stiffness or elastic modulus has been increasingly reported in 3D bio-tissue studies. The substrate stiffness, topography, rigidity, immobilized and soluble signals affect cell adhesion, differentiation, migration, and proliferation through the focal adhesion-cytoskeleton dynamics, consequently affecting cell behaviors and fates [11]. The biomechanical testing methodologies of 3D samples applied in the field varied based on many factors, including and not limited to the research questions being investigated: the scale of samples, nature of the characterization and type of testing, equipment availability, and sufficient sensitivity. For example, short-term traction force measurements of individual cells cultured in 2D format on hydrogels would benefit from 2D traction force microscopy [12–14] or 3D traction force microscopy techniques [15]; while cellular tractions cultured in a 3D context within matrices would benefit from a similar approach of 3D traction force microscopy that is also based on embedded microbeads [16]. The optical tweezer also has its advantages, such as high sensitivity and disadvantages, such as the small measurable/applicable force ranges [17], photodamage, lack of selectivity, and exclusivity. Shear/flow-based microfluidics are typically used for targeted applications [18]. Rheology testing is widely used for hydrogel characterization. When considering rubber elasticity theory [19], where rubbery shear modulus G is independent of the frequency, measurements can be made in a low-frequency mode, assuming quasi-static deformation. However, microscopic heterogeneities observed in a variety of hydrogels are lost in this bulk measurement.

Atomic force microscopy (AFM) has many versions developed over time, where each modality accommodates certain operation features with extremely high resolution. The reader is directed to the detailed review of AFM imaging modes [20]. Considerable efforts were made to standardize nanomechanical AFM procedure (SNAP) for soft and biological sample measurements, where they reported reduced variability (1%) in hydrogel elastic moduli evaluation and increased consistency in elasticity measurements by a factor of two [21]. However, since AFM measurements are extensively utilized for localized tissue and mechanical cell measurements [19, 22], they have been reported to be less suited [23] for bulk mechanical characterization of collagen hydrogels [19] and larger structures including multicellular spheroids [24]. AFM-based cantilevers were tested with several instruments for different applications and testing conditions [20], with various spring constants and probe dimensions and applicable contact models [25].

Some tools have recently become commercially available for mechanical testing of 3D tissue cultures. Chondrospheres (spheroids of chondrocytes) have been mechanically characterized using micro-scale parallel-plate compression testing, Microsquisher (CellScale) [26]. Another tool (Pavone by Optic11) is commercially available for mechanobiological screening in 96 well-plates; this system is based on a glass-fiberoptic cantilever bending and force sensing. However, the Pavone operates based on a single cantilever which must go in and out of every well; concerns relating to open-chamber, potential cross-contamination, and repeated bubble generation between wells may dramatically affect the efficiency of testing time.

Our group has reported micromechanical tweezer systems, where spin-coated photopolymer (SU-8)-based cantilevers in a micro tweezer format were used to analyze the stiffness of healthy and cancer breast spheroids directly. Moreover, agarose hydrogel pillars of various concentrations were tested, and the approach was neatly validated with ball indentation measurements [24]. However, SU-8 is not an FDA-approved material for biological applications, and microcantilevers may easily break during handling. Spin-coated elastomer (polydimethylsiloxane (PDMS))-based cantilevers were reported for the stiffness monitoring of zebrafish embryos during embryonic development stages [27]. Both reported micro tweezer systems had low-profile designs to conduct side compression testing of biological samples using brightfield inverted microscopes while allowing testing under a confocal microscope as well. However, the side tweezer approach may hinder efficient arrayed sample testing in the same petri dish and further complicate the design of sterile closed-chamber long-term testing, which may add the material supplies, time, and cost needed to conduct such experiments.

### 1.2. This study’s approach

This study reports the design, fabrication, and testing of our novel PDMS-based micro tweezers (μTweezers). The tweezer system fits on top of Petri dishes/culture plates on an inverted microscope for efficient 3D arrayed organoid/tissue mechanical characterization. The design aims to boost the technologies to test biological samples in the micro-mesoscale range to match the increasing demand for mechanical testing of 3D tissue cultures. The μTweezer system can mechanically characterize 3D tissue cultures of defined structures, including and not limited to hydrogel pillars, organoids, spheroids, tumoroids, chondrospheres. The system fabrication relied on the widely available milling and molding techniques [28] rather than previously reported spin-coating approaches [24, 27]. The 3D elastomer molding technique allowed the fabrication of mechanically functional 3D PDMS devices with sub-millimeter-scale resolution. The manipulator interface allowed the transfer of forces from external precision micro-actuators to PDMS-based end-effectors (cantilevers), delivering micro-indentations to the bio-samples. The deflections of the calibrated cantilevers were measured and analyzed through microscopic observation to acquire the stiffnesses of the sample groups.

To demonstrate the efficacy of the μTweezers, we created arrayed organoid pillar samples from bioactive collagen I hydrogels embedded with an ovarian cancer cell line (SKOV-3) as a 3D *in vitro* model to study human ovarian cancer [4, 29]. Trending 3D tissue culture models for cancer cells include but are not limited to spheroids and organoids, either hydrogel-free or hydrogel-based [4, 30]. Such models have been utilized to study the cellular behavior in response to the 3D matrix or hydrogel environment, which is believed to represent the original tissue characteristics [31].

There is a growing demand for user-friendly, accurate mechanical characterization devices to be used with standard lab equipment and tissue culture ware while maintaining high force sensitivity and long-term biocompatibility. Many of the available high-end technologies rely on delicate alignment and calibration, requiring intensive user training to get accurate and reliable data, which may deter the progress in the growing field. In addition, customizations to existing high-end equipment may not always be financially justifiable or the preferred route. Our system’s properties can be easily tuned and customized to accommodate the sensitivity, spring constant, and sizes of different 3D models. The platform is biocompatible for long-term use, which provides an attractive tool in biomedical and biochemistry fields, including regenerative engineering, personalized medicine, and biomaterials sciences.

## 2. Materials and methods

### 2.1. Device design, fabrication, and testing

#### 2.1.1 System design

**Fig 1:**
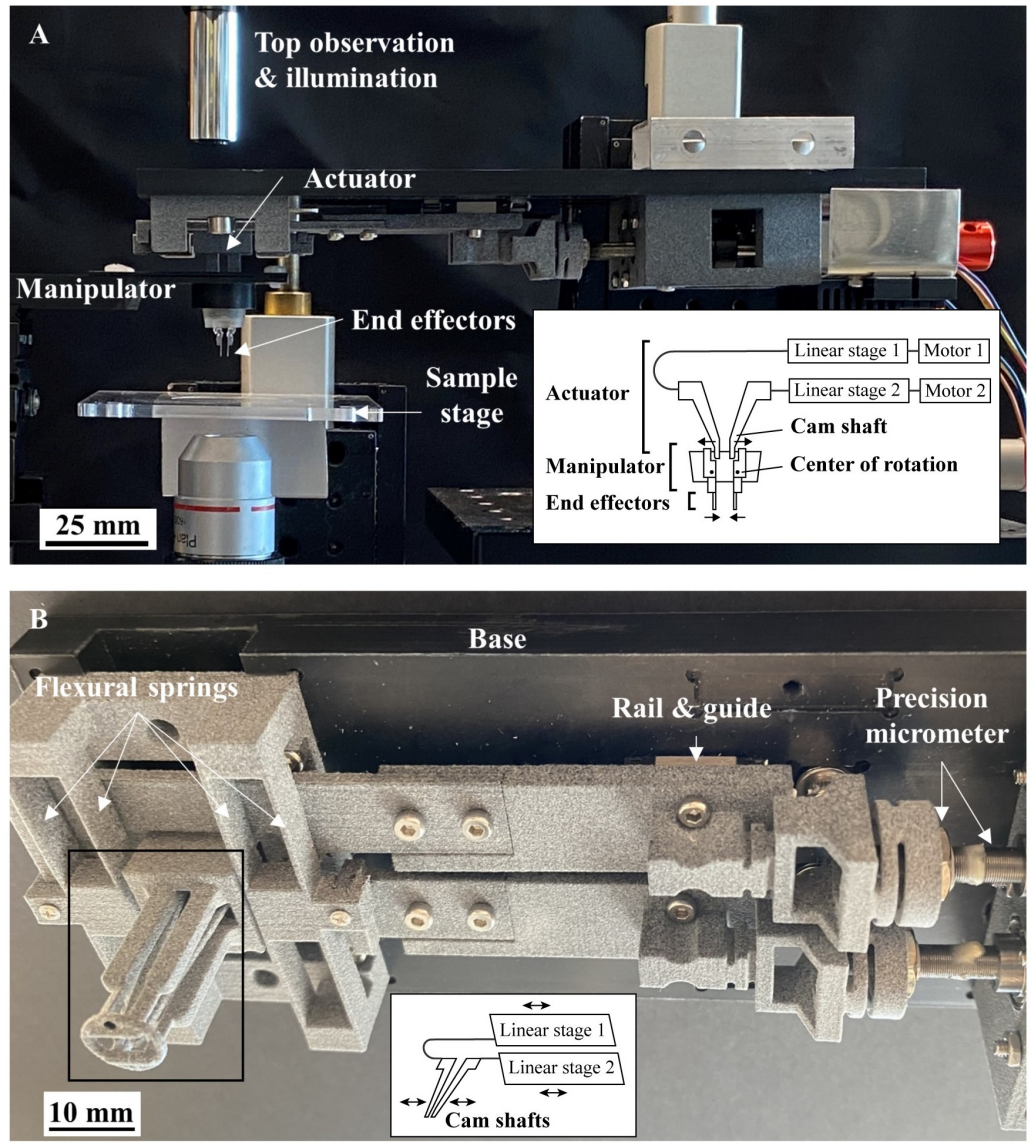
(A) Platform assembly showing subassemblies, (B) precision micrometer actuator subassembly and components. Further details of the actuation mechanism are illustrated in Fig 2.

**Fig 2:**
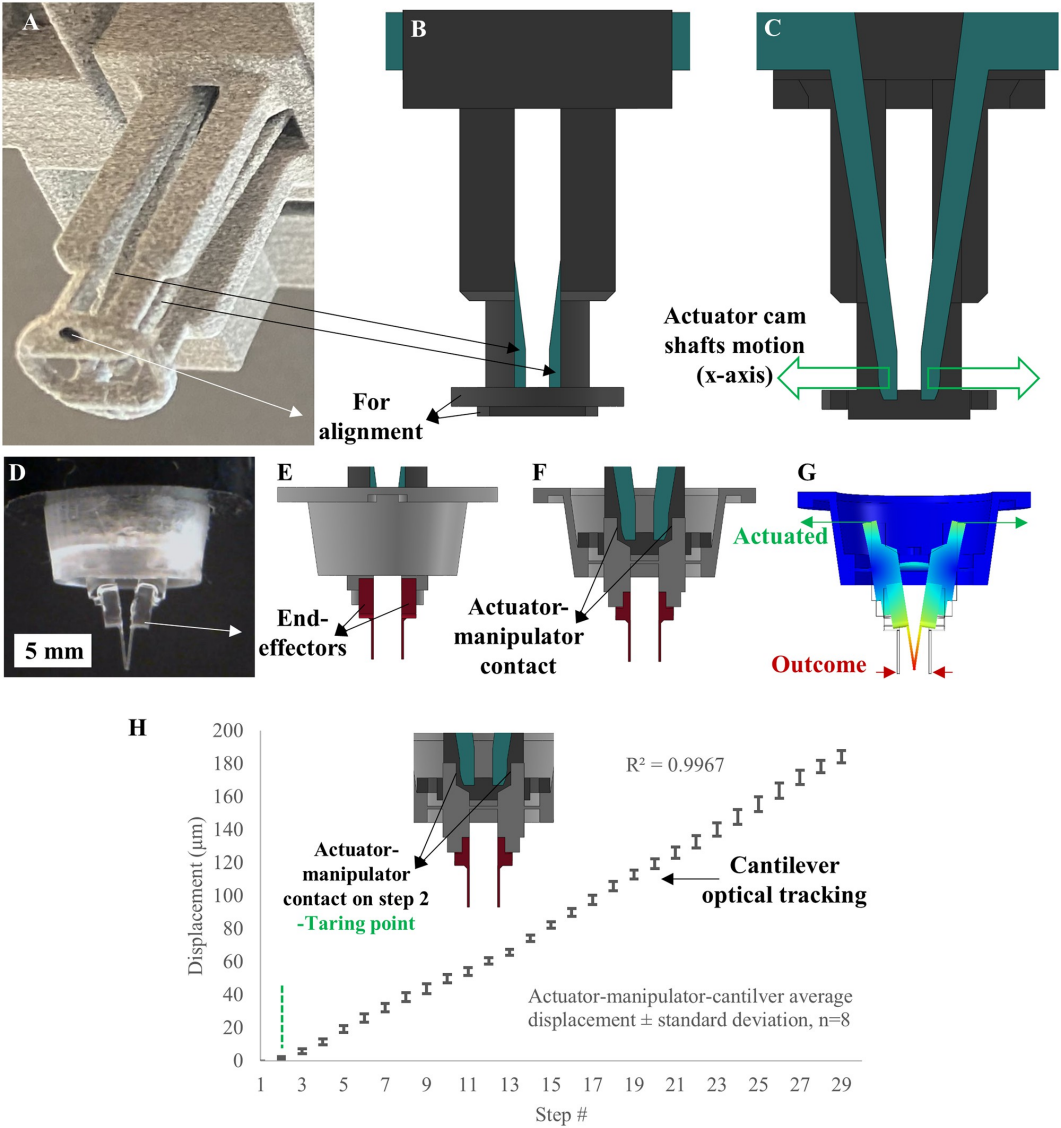
Motion transfer interface, (A) a subset of the actuator tower; (B) a side view of the actuator tower; (C) a cross-section side view showing the camshafts in green and their X-axis motion in arrows; (D) actual assembled device, (E) a side view of the assembly, manipulator(grey) and end effectors (red); (F) a cross-section side view of the assembly showing the actuator to manipulator contact, where camshafts are shown in (green), manipulator in (grey), and the end effectors in (red); and (G) a cross-section side view COMSOL simulation showing the delivered motion profile of the actuation. (H) Actuator-manipulator-end effector repeated motion performance (with no sample). The delivered motion linearity is tared at step 2 (R^2^ = 0.99).

The overview and the platform assembly are shown in Fig 1. The system has three main subassembly sections:

1. **Actuator**. A custom-built precision positioning system (Fig 1A) consisting of an Arduino controller, electronic motor drivers, stepper motors, couplers, fine adjustment screws, externally threaded nuts (Thorlabs Inc., Newton, NJ, USA), 3D printed flexure-based actuator camshafts (Shapeways Inc., New York, NY, USA) fixed on a machined ABS plate (Mcmaster carr, Elmhurst, IL, USA). The camshafts (framed in Fig 1B) get lowered into the manipulator, while the motion of the actuator tower and camshafts is further explained in the following figure.
2. **Manipulator**. A cup-shaped insert made of PDMS. It includes thin membranes, which work as the interface to transfer the motion and force from the actuator camshafts to the micro end-effectors (cantilevers). The manipulator thin membranes (~50μm) are permeable to CO_2_ [32] and provide transparency for top illumination.
3. **End-effectors**. The end effectors are cantilevers made of PDMS. The cantilevers’ typical dimensions were 2.8 mm (length) × 800 μm (width) × 50 μm (thickness) for 10X imaging magnification. The actual measured dimensions are discussed in the cantilever design section.

The system is compatible with 35 and 60mm Petri dishes (open chamber for endpoint tests) and 24-well tissue culture plates (closed chamber for time-course experiments). The platform operates in combination with an inverted microscope consisting of precision x-y-z stages, CMOS camera (Chameleon3 CM3-U3-50SM, FLIR Systems Inc., Wilsonville, OR USA), and a 4X or 10X objective lens (United Scope LLC dba AmScope, Irvine, CA, USA), a computer installed with ImageJ (Version 1.52a, National Institute of Health, Bethesda, MD, USA) and MATLAB (Version R2019a, Mathworks, Natick, MA, USA).

Fig 2 shows a detailed view of the motion transfer interface between the camshafts and the manipulator. The two camshafts are actuated by the stepper motors (Fig 2A–2C). The cams push the elastic hinges supported by a membrane built in the manipulator (Fig 2D–2G). The platform performance and linearity rely on the accuracy of the camshafts’ positioning and contact to the PDMS manipulator interface. The cantilever positioning accuracy was measured, plotted, and explained in Fig 2H, while the cantilever-sample contact is detailed in the measurement section.

#### 2.1.2 Manipulator fabrication

**Fig 3:**
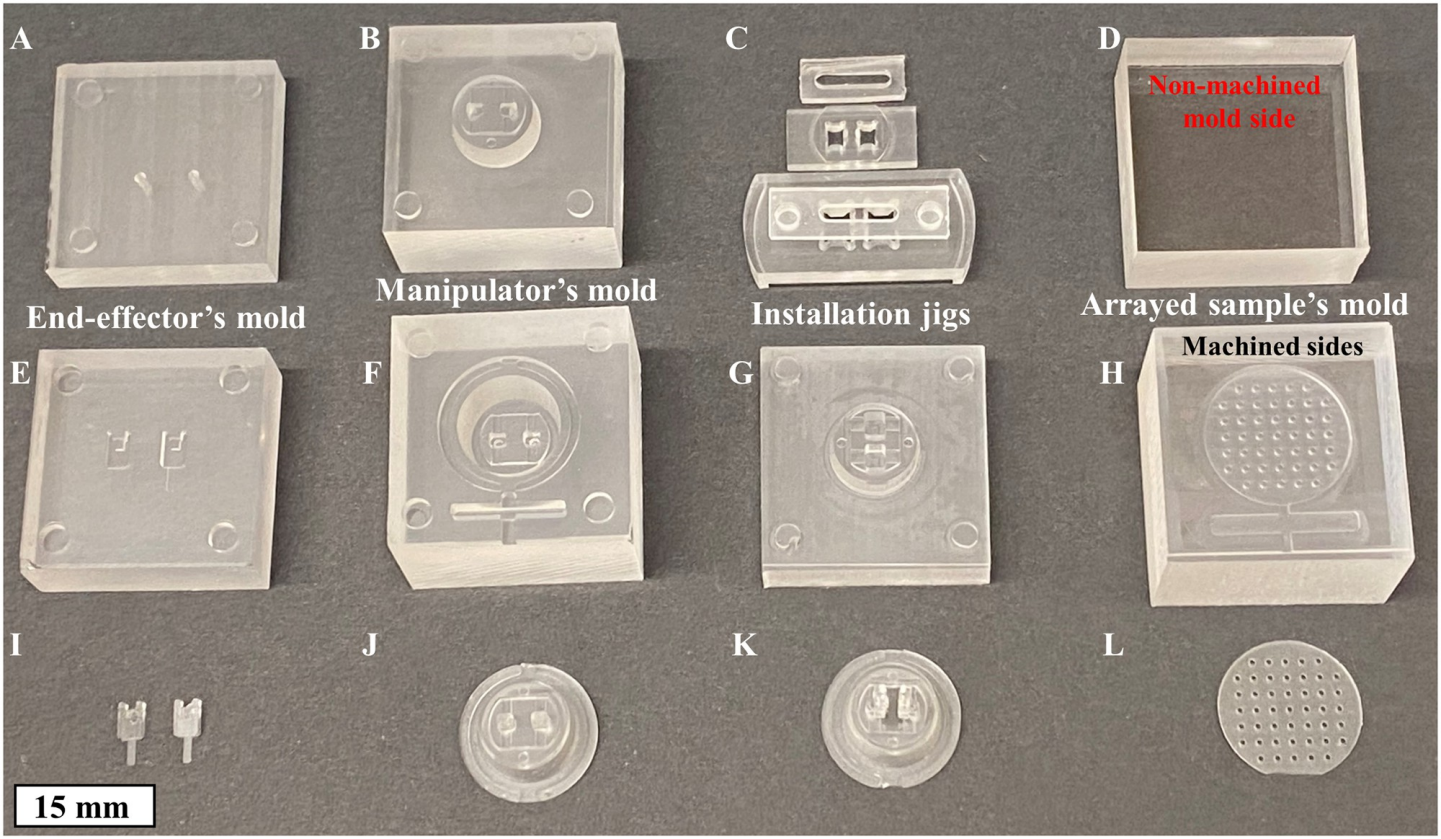
(A-H) micromachined acrylic molds for PDMS parts and installation jigs as labeled; and (I-L) are the cured PDMS outcomes of the parts above it. The cantilevers (I) and manipulator (J) are assembled into a single piece (K), while (L) shows the arrayed PDMS mold used for hydrogel arrayed pillar generation. The whitish face shown in the image (L) is the side that was in contact with the machined side of mold (H).

Acrylic (PMMA) blocks obtained from (Mcmaster carr, Elmhurst, IL, USA) were machined using a high-resolution CNC milling machine (monoFab SRM-20, Roland DGA Corp., Irvine, CA, USA), creating fine 3D structured molds to be used for PDMS casting/molding (Fig 3). Similarly, acrylic jigs were machined for the end-effectors’ installation. This jig is used when the cantilevers are aligned and glued to the manipulator aided by precision-guided mechanical stages. A drop of PDMS was used as glue, and the jig held the cantilevers in place during curing.

The elastomer molding technique allowed easy and accurate fabrication of mechanically functional 3D PDMS devices with a micro-scale resolution. The PDMS (Sylgard 184 Silicone elastomer- Dow Corning Corp., Midland, MI, USA) base and curing agent mixtures were prepared at a 10:1 ratio, respectively. All PDMS parts throughout this study used the same mixing ratio, as suggested by the manufacturer. Degassing took place in a desiccator for 15 minutes (Bel-Art Products, Inc., Wayne, NJ, USA). The molds were then sandwiched, vise clamped, and placed in a convection oven at 65 C° for 12 hours curing time. In this study, the elastic modulus of the PDMS cantilevers was calibrated and measured (2.46 MPa) [28]. Only the PDMS molds in (Fig 3L) were cured for 3 hours at 65 C°.

Another PDMS stiffness refining approach would be by changing the PDMS curing conditions without changing the geometry. Increased hardness can benefit some PDMS parts of the assembly (e.g., the manipulator or stiffer end-effectors). However, excessive crosslinking curing agent ratios were avoided to avert the potential of excess crosslinking agent leaching out into cell culture and maintain high PDMS elasticity, which also supports the PDMS actuation and bending with reduced to no hysteresis. Further relevant fabrication and curing condition details were previously reviewed and reported [28].

#### 2.1.3 Cantilever design and calibration

The cantilever formulas allow easy tuning/refining of the geometry. Therefore, it is easy to tune the spring constants based on the required specifications, including sample size, linear range & sensitivity. Our dimensions (*L* = 2.8 mm, *W* = 800 μm, and *T* = 150 μm) were designed to measure soft stiffness ranges which were adequate and applicable to the collagen-based samples used in this study. The following equation gives the spring constant of a cantilever:

$$K=\frac{EI}{L³}$$ (1)

Where (*E*) is Young’s modulus of the cantilever, and (*L*) is the cantilever length. For rectangular cantilevers, the second moment of area (*I*) is given as

$$I=\frac{WT³}{12}$$ (2)

using the cantilever width (*W*) and thickness (*T*). The Eq (1) can be substituted and rewritten as:

$$K=\frac{EWT³}{4L³}$$ (3)

Based on the design dimensions and the elastic modulus *E* = 2.46 MPa we found in our previous study [28], the calculated PDMS cantilever spring constant was (0.0765 N/m), and COMSOL stationary solid mechanics’ simulation resulted in a spring constant (0.08 N/m). The spring constant calculated using the measured average cantilever thickness (148 ± 1.9 μm, n = 8) was (0.0725 N/m), and the updated COMSOL spring constant became (0.079 N/m).

Before using, we calibrated the cantilever spring constant using a reference cantilever (RRC) with a known spring constant (0.151 N/m). The detailed method of the cantilever calibration was previously reported by our group [27]. Based on Hook’s law, an applied force results in a linear elastic deformation as follows:

f=Krdr=Kcdc (4)

Where (*f*) is the force, (*Kr*) is the RRC spring constant, (*dr*) is the RCC displacement, (*Kc*) is the spring constant of the PDMS cantilever, and (*dc*) is the PDMS cantilever displacement. The calibration resulted in a spring constant (0.0718 ±0.0073 N/m, n = 3, R^2^ = 0.98). In our system, the end-effectors work both as an indenter and a sensor simultaneously. By knowing the spring constant, the optically measured end-effector’s displacement represents the linear applied forces.

#### 2.1.4 Manipulator sterilization

The PDMS manipulators, end-effectors, and sample molds were soaked with Isopropanol alcohol 70% for 20 minutes, rinsed with sterile water, and dried in a sterile biosafety cabinet. Autoclaving PDMS parts was avoided because the heat can dramatically change the spring constant and device performance between different sterilization cycles and affect stiffness measurements.

### 2.2. Biosample preparation

#### 2.2.1 Collagen I hydrogel neutralization (all steps chilled on ice)

Nine parts of Collagen I solution from bovine skin (C4243, Sigma-Aldrich, St. Louis, MO, USA) were added to a 15 ml tube containing one part of the neutralizing solution (5229, Advanced BioMatrix, Inc., San Diego, CA). The pre-formulated neutralizing solution is a 10X PBS that has been pH adjusted using basic NaOH. For consistency with hydrogel-based organoids that included cells and to get a softer hydrogel range, one part of the neutralized hydrogel was diluted with two parts media, either with cells or without cells, which roughly further diluted the collagen I concentration to ~1.86–2 mg/ml.

#### 2.2.2 Hydrogel pillar array preparation protocol

**Fig 4:**
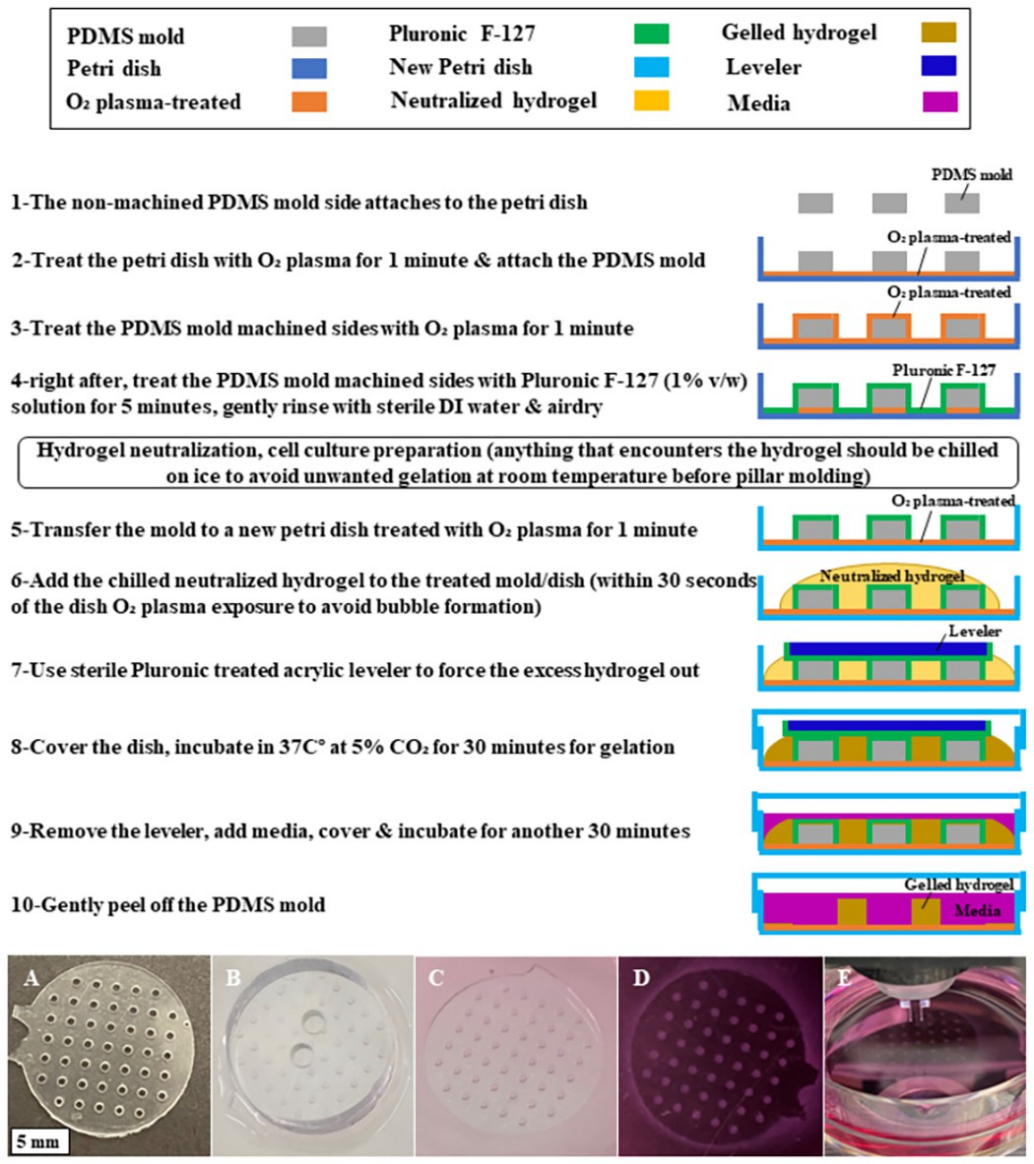
(Steps 1–10) Hydrogel casting/molding protocol. (A) the PDMS mold showing the non-machined, shiny side; (B) acrylic leveler which had a smooth surface and sufficient weight was used to force excess hydrogel to the sides (step 7); (C) After the leveler was removed, media was added (step 9); (D) PDMS mold was the peeled off, and arrayed hydrogel pillars are ready for mechanical testing shown in (E).

The preparation steps of the hydrogel pillar array are shown in Fig 4, followed by actual images representing the success of steps. Fig 4 illustrates the preparation of the hydrogel pillar array, which utilized the PDMS mold (see Fig 3L) with a fixed pillar geometry. The pillar was sized 750 μm in diameter and 500 μm in height, while the PDMS mold diameter was 15 mm. Each mold has 45 pillars with sufficient distance in between to allow for distances between samples. The hydrogel molding protocol in Fig 4 was inspired by a suspended hydrogel pillar protocol [33]. Our study further modified the protocol to fix the hydrogel pillars to the petri dish for efficient array testing.

The PDMS mold was surface-functionalized using an oxygen plasma cleaner (PDC-32G, Harrick Plasma Inc., Ithaca, NY, USA) for 1 minute at 18W power (high RF) and a Pluronic F-127 solution (1% v/w, P2443- Sigma-Aldrich, St. Louis, MO, USA) in steps 1–4. After the neutralization of hydrogel, time-sensitive steps of surface treatment and hydrogel casting, gelling, and leveling (steps 5–8) must be conducted promptly. The side of the non-machined PDMS mold is smooth and shiny, while the machined side has a rougher surface. The non-machined (shiny) side should always be used as the side that attaches to the petri dish to prevent the Pluronic F-127 solution from leaching between the PDMS mold and the petri dish. Using the smooth side for attachment is also essential to avoid slipping the mold, which would dislocate the pillars from the dish.

#### 2.2.3 Cell culture

For stiffness analysis, a human ovarian adenocarcinoma cell line SKOV3 was used, which was derived from the ascitic fluid of a 64-year-old Caucasian female with an ovarian tumor (91091004, Sigma-Aldrich, St. Louis, MO, USA). They were cultured on treated cell culture flasks in 2D monolayers in McCoy’s media (McCoy′s 5a, 2mM Glutamine, and 10% Fetal Bovine Serum FBS) and changed every 2–3 days. At 70–80% confluence (passage 5), they were rinsed with PBS, suspended using 0.05% trypsin, incubated for 4 minutes, pelleted by centrifuging at 1000 rpm for 5 minutes, resuspended in a fresh medium using a tube shaker for twenty seconds. The cells are then seeded into neutralized collagen I hydrogels at a concentration (1.5×10^6^ cells/ml) a couple of minutes before hydrogel casting into the PDMS molds. On average, 250–350 cells per organoid were achieved.

Adult Human Dermal Fibroblasts (HDF) (HDF-1N55+, Cascade Biologics, Portland, OR, USA) were used for viability tests at 80–90% confluence (passage 14) in a cell culture medium including (Dulbecco’s Modified Eagle Medium DMEM, 1% GlutaMAX, 1% Sodium Pyruvate, 10% Fetal Bovine Serum FBS, 1% penicillin-streptomycin (10,000 U/mL)). Cells were rinsed with PBS, suspended using 0.25% trypsin, incubated for three minutes, pelleted for 5 minutes using a centrifuge at 1200 rpm, resuspended in a fresh medium using a tube shaker for twenty seconds, and seeded on the 24 well plates with media change every 2–3 days for 3 weeks. (PBS, DMEM, GlutaMAX, Sodium Pyruvate, FBS, penicillin-streptomycin, trypsin, McCoy′s 5a, and Glutamine were sourced from gibco, via Thermo Fisher, Waltham, MA, USA).

#### 2.2.4 Cell viability

**Fig 5:**
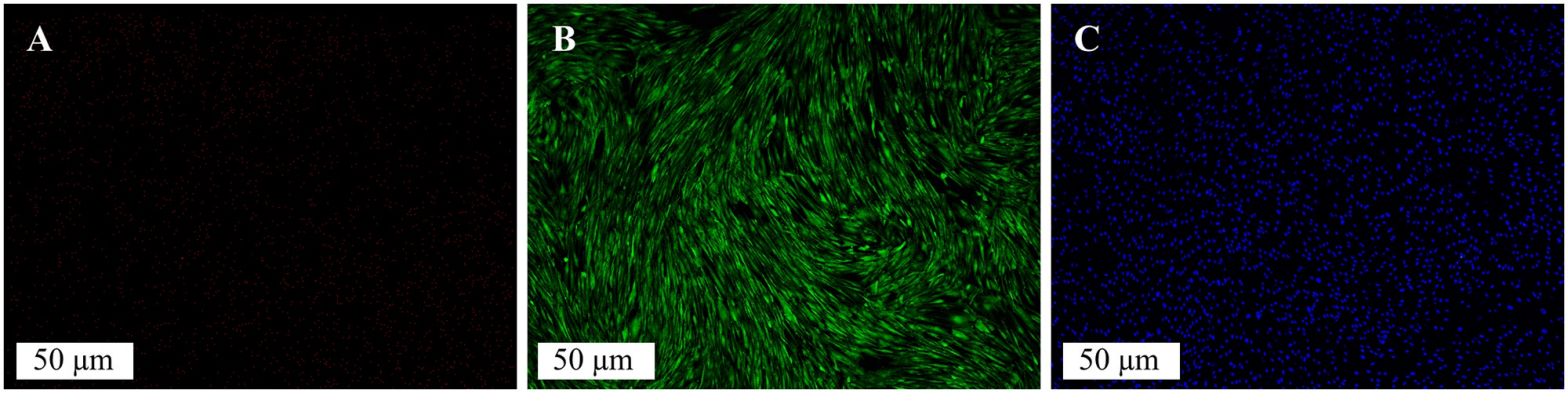
The viability of HDF in a 24 well-plate and stained for fluorescence observation after cultured in monolayers for three weeks in a chamber closed with the PDMS manipulator. (A) the dead cells in red (Texas red filter), (B) the live cells in green (FITC filter), and (C) stain bound to HDF nuclei DNA (DAPI).

To evaluate the systems’ biocompatibility without 3D cell culture effects such as necrosis and hypoxia, 2D monolayers of human dermal fibroblasts were cultured in 24 well-plates with the PDMS manipulators, starting at low seeding numbers and achieving high numbers over time. Our previous publication [28] tested PDMS characteristics under a cell culture condition and demonstrated biocompatibility using the human dermal fibroblast cell line. This study used the same cell line to compare with the previous well-tested PDMS device design and use the results as a reference. After three weeks of culture, cells were rinsed with PBS and stained with live/dead assay solution at room temperature for 30 min and NucBlue (DAPI) for nuclei staining (L3224, R37606, Invitrogen, Thermo Fisher, Waltham, MA, USA), respectively. The viability and nuclei of cells were observed under a fluorescence microscope (Olympus BX51, Olympus Corporation of the Americas, Center Valley, PA, USA) to show the systems’ biocompatibility in the closed chamber version. The PDMS device was highly biocompatible, and we did not observe dead cells in this testing (Fig 5).

### 2.3. Force sensing, displacement measurement, & analysis approach

#### 2.3.1 Force sensing

**Fig 6:**
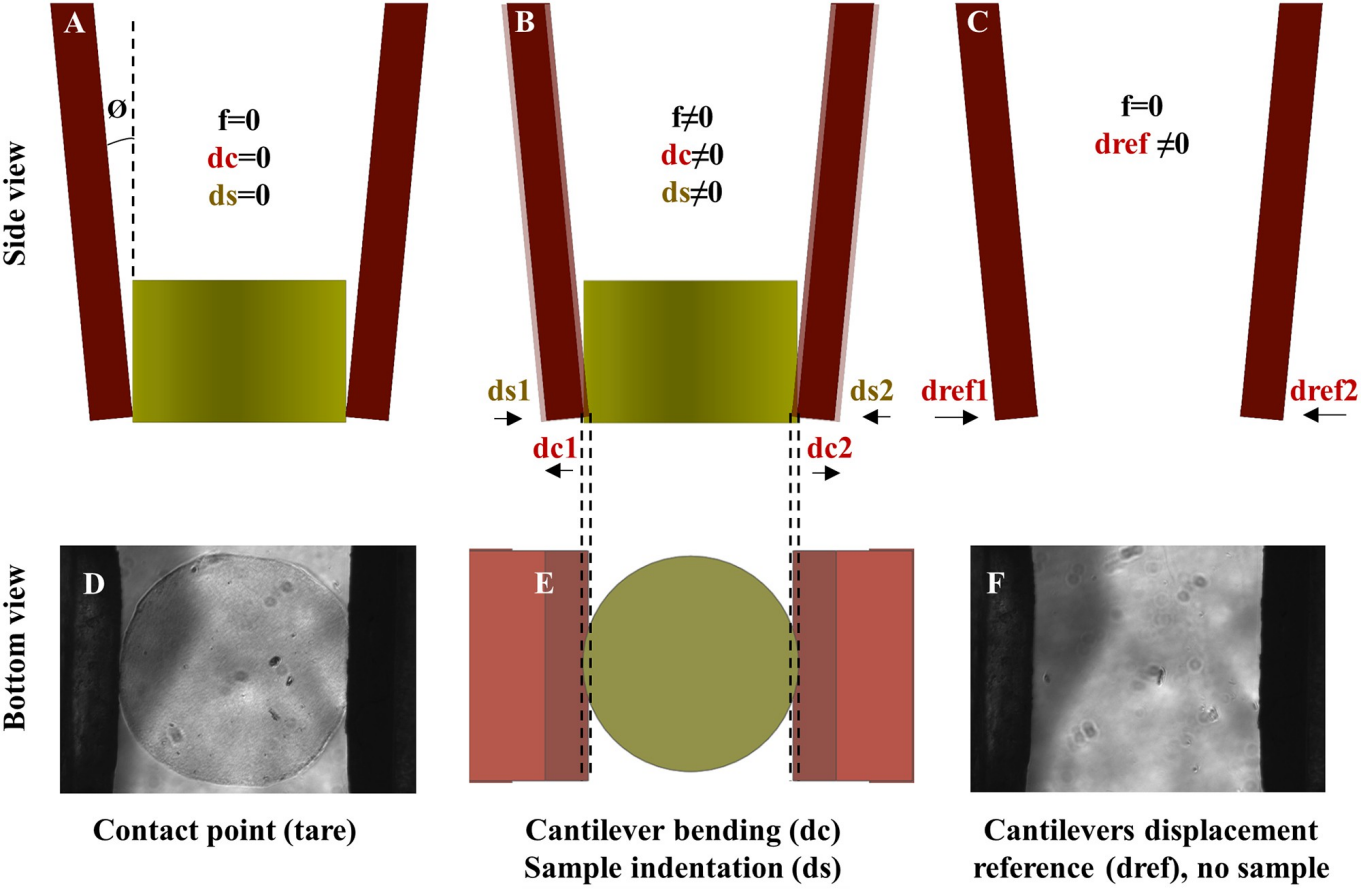
The end effectors (cantilevers) deflections with and without a sample; (A-C) sides views of (A) the contact point, (B) the cantilever bending (with a sample); and (C) the cantilever displacement (without a sample). (D-F) bottom views of (D) the contact point, (B) the cantilever bending (with a sample), and (C) the cantilever displacement (without a sample).

The μTweezers testing approach relies on the cantilever deflections; the differences of cantilever deflections with or without samples give the sample indentation and the cantilever bending (Fig 6). The sample indentation is tared at the sample contact point (Fig 6A). The data was recorded up to a targeted strain limit during indentation to ensure the measurements were in the linear range. In this study, 4% of the diameter was applied as the strain cut-off limit. The approach used in this study follows the steps as follows:

1. The measurement starts once the end effector touches the sample (i.e., the parameters are tared at the sample-end effector contact point).
2. With a sample, images were taken after each step in a quasi-static manner, which provided tiles of images displaying the displacements of the cantilevers’ deflections (*dc*) indenting the samples by (*ds*).
3. Without a sample, the same process was repeated but without a load (force = 0) to have reference displacement (*dref*) profiles of the cantilevers.

When a sample is placed between the tweezer cantilevers, the force across the sample during compression based on Hook’s law was described as:

$$f_{1}\;\text{cos}(\theta_{1})={kc}_{1}\;{dc}_{1}$$ (5)

$$f_{2}\;\text{cos}(\theta_{2})={kc}_{2}\;{dc}_{2}$$ (6)

Where (*f*) is the force, (*kc*) is the cantilever spring constant, and (*dc*) is the cantilever bending (with sample).

Ideally, the cantilever alignment and bending are symmetric (*θ*_1_ = *θ*_2_, *dc*_1_ = *dc*_2_ = *Dc*), and the cantilever spring constants are the same (*kc*_1_ = *kc*_2_ = *Kc*), which results in (*f*_1_ = *f*_2_ = *f*). We found the force (*f*) by using the averaged cantilever bending $Dc=\frac{{dc}_{1}+{dc}_{2}}{2}$. We also assume the same cantilever orientation *θ* from start to stop (5.28°-5.43°), which is adequate because (cos (5.28°) = 0.9957, and cos (5.43°) = 0.9955), respectively. Thus, the force (*f*) was now simplified to:

fcos(θ)=KcDc (7)

Following every sample, a reference run recorded the displacements between the cantilevers without a sample (*dref*), which was used to calculate the sample indentation (*ds*) where:

dref=dc+ds (8)

Given that the sample indentation data was used only in the linear range, and the measurements conducted were in a quasi-static manner, it is safe to assume that the sample acts as a linear spring having a spring constant (*Ks*).


f=DsKs (9)

Using the force from Eq (7) and the averaged sample indentation $Ds=\frac{{ds}_{1}+{ds}_{2}}{2}$, Eq (9) gives the sample stiffness or spring constant (*Ks*) as:

$$Ks=\frac{f}{Ds}=\frac{kc\;Dc}{Ds\;\text{cos}\;\theta \;}$$ (10)

#### 2.3.2 Cantilever-sample indentation measurement

**Fig 7:**
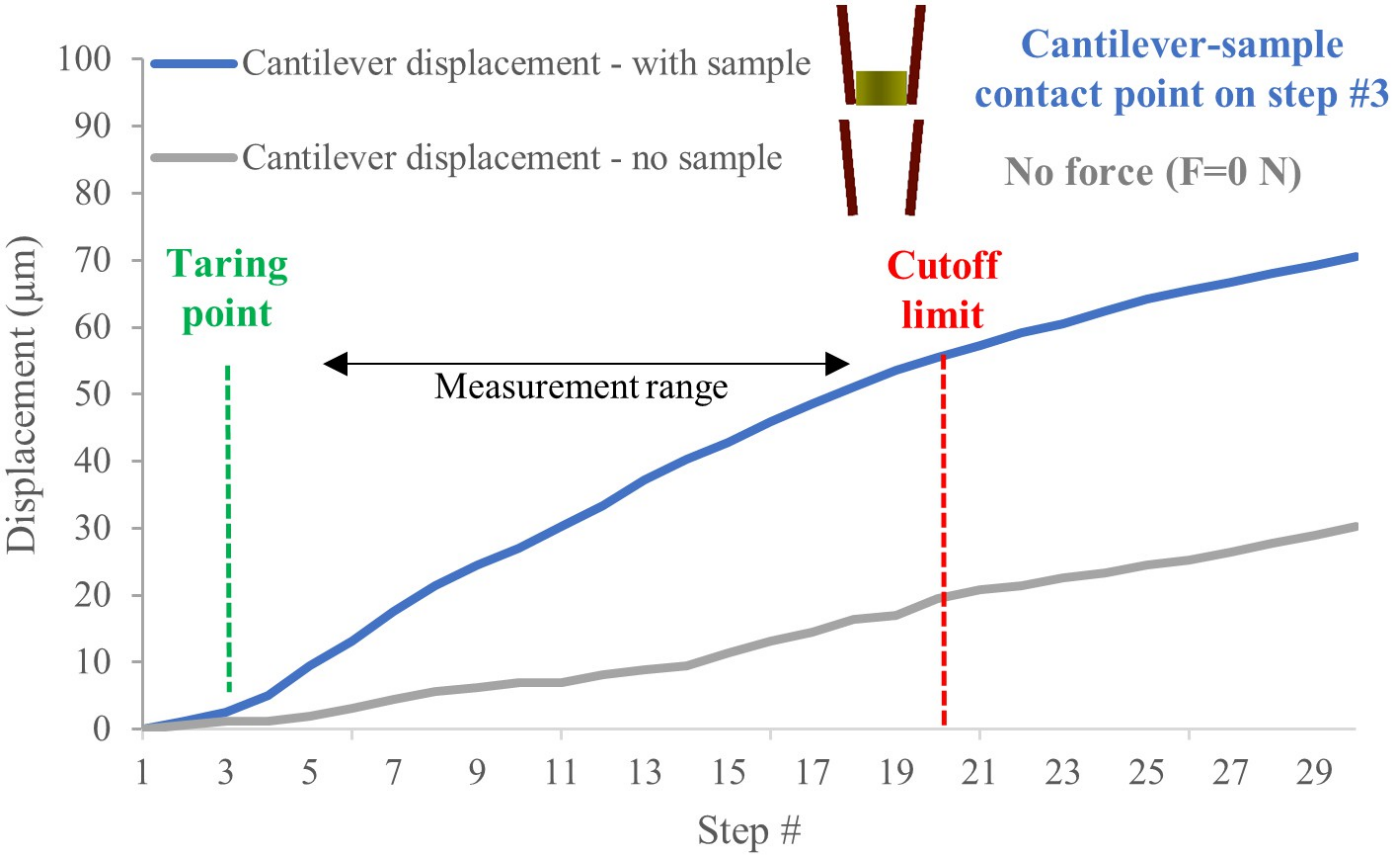
Finding the cantilever-sample contact and measuring the following indentation. Once the cantilevers engage with the sample, a change in the slope is observed and recorded. In this measurement, Step 3 (Taring point shown in green) was the sample contact point where the displacement measurement started. Step 20 (red) was the cut-off strain limit considering the end-effectors’ calibration linear range and the targeted strain applied to samples.

After the actuator–manipulator initial contact (discussed previously in Fig 2H), a sample is placed between the cantilevers. As the cantilevers approach the sample, the displacements profiles are tracked through microscopic imaging. Another crucial point is the contact between the cantilevers and the sample, which must be carefully considered and monitored for accurate displacement measurements (see Fig 7).

#### 2.3.3 Image tracking and data extraction

The scale (pixel/μm) of the microscope images was obtained using ImageJ and an optical scale. Custom-programmed MATLAB codes were used to track the end-effector displacements, with a sample (*dc*) or without a sample (*dref*), by tracking the displacement pixels of an area of interest through the image tiles of compression steps. The sample indentation (*ds*) was calculated as the difference between the contact point and the current position Eq (8). Using the same manipulator and end-effectors for consecutive measurements did not affect the accuracy as every measurement was directly followed by a reference measurement (with no sample).

#### 2.3.4 Parallel plate compression (PPC), mechanical testing (reference testing)

To find the reference elastic modulus, we tested the same hydrogel group (Control at day 0, n = 4) using a typical parallel plate compression (PPC) at strain 4% in a quasi-static manner at the same strain rate used in the tweezers setup (1.25 μm/sec). The elastic modulus of the hydrogel discs was obtained using a hydrogel pillar with dimensions of (radius = 5 mm and height = 2 mm) and the simple linear cylinder compression formula:

$$\frac{F}{A}=E∙d\;L$$ (11)

where (*A*) is the cylinder cross-sectional area A = *πr*2, (*E*) is Young’s modulus, and *d L* is the compression length. As the elastic modulus or Young’s modulus (*E*) is a material property and not a dimension property, the measured Young’s modulus (*E*) of the group (Control at day 0) was applied to the same group using the micro tweezers. The other groups’ elastic moduli were calculated relative to this reference value based on the hydrogel measured stiffness or spring constant (*Ks*).

### 2.4 Statistical analysis

ANOVA and student t-tests were used for the statistical data analysis where data is reported as average ± standard deviation unless otherwise stated. The number of samples and linearity fitting (R^2^) are included when applicable. p-values of less than 0.05 (p-value < 0.05) were considered statistically significant and assigned (*).

## 3. Results

### 3.1. Organoids’ stiffnesses

**Fig 8:**
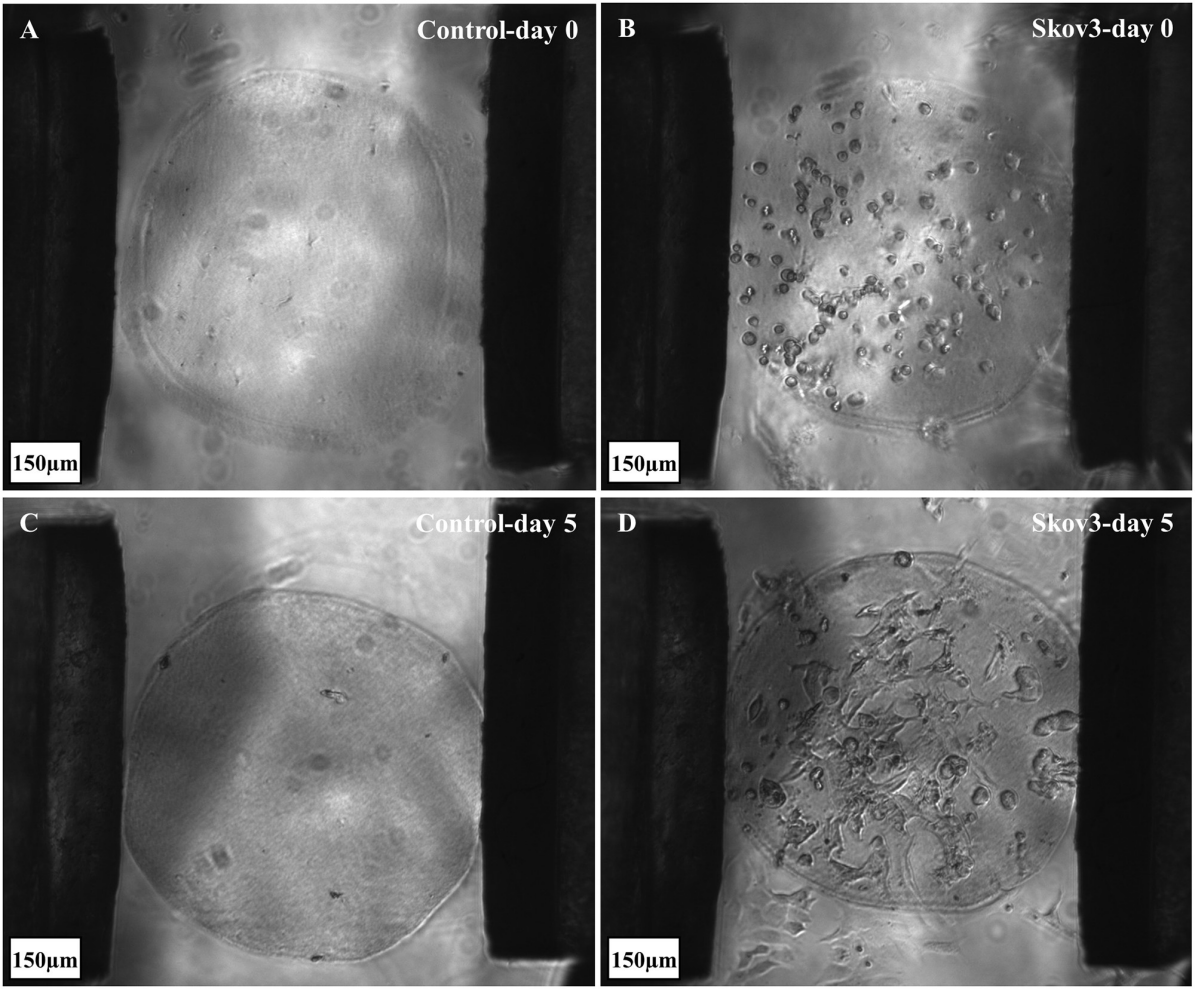
The four tested groups of hydrogel organoids, (A) control—day 0, without cells; (B) SKOV3 embedded organoids- day 0; (C) control—day 5; and (D) SKOV3 embedded organoids- day5.

**Fig 9:**
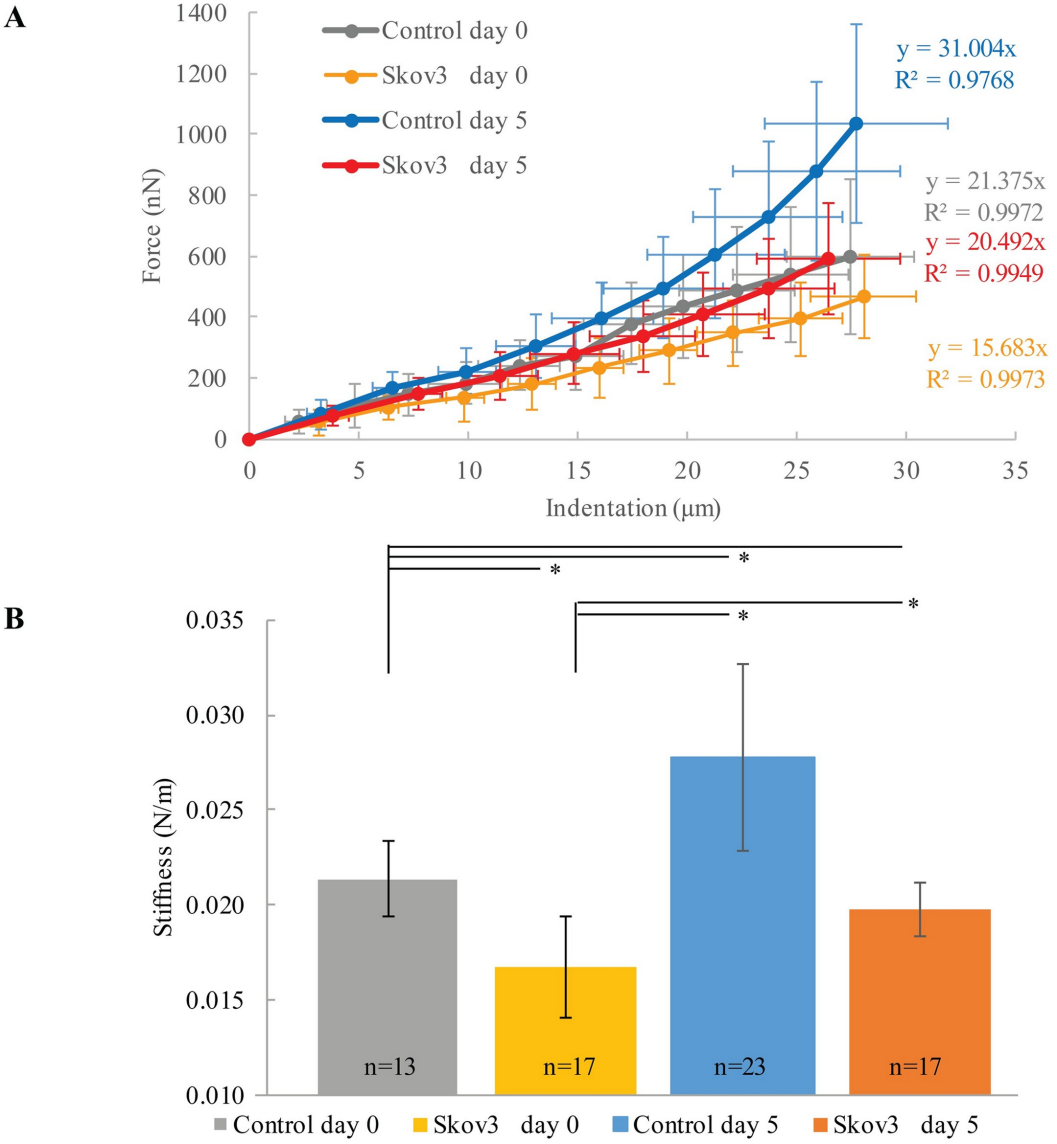
Stiffness measurements across the four groups, (A) displays the force curves, and the average measured stiffness in (nN/μm) with standard deviations plotted in linear fittings; (B) the four groups’ stiffness in (N/m) with statistical analysis, where (p-values < 0.05) were assigned (*).

The stiffness of the hydrogel-based organoids was measured for four groups using the micro tweezers, namely, Control at day 0, ovarian cancer cells SKOV3 at day 0, Control at day 5, and SKOV3 at day 5 (Fig 8). The results show significant increases in stiffness from day 0 to day 5 in both Control and ovarian cancer embedded organoids (Fig 9). Moreover, when comparing the stiffness of controls to the stiffness of cell embedded organoids, both at day 0 and day 5, significantly lower stiffnesses for groups with embedded cells were measured. The diameter changes across groups are explained in the following sections.

### 3.2. Organoid diameter shrinkage

The designed pillar diameters were fixed at 750 μm; however, the initial reference group (control—day 0) had a (~5.85%) reduction in diameter right after gelling because of the variation caused during manual fabrication and the shrinkage through hydrogel solidification (polymerization/gelling).

We also observed shrinkage through the five-day experiment. The observed results showed (<5%) reduction in diameter for groups (SKOV3—day 0) and (control—day 5) compared to the reference group (Control—day 0), while the last group (SKOV3—day 5) had 9.1% decrease in diameter indicating more cellular shrinkage over time. SKOV3 cells in the hydrogel-based organoids increased the shrinkage compared to the control group by (3.95%) on day 0. The lowest reduction was between controls at days 0 and 5 (2.78%), while the most shrinkage (9.1%) occurred in the group (SKOV3—day 5). A (6.32%) shrinkage in the group (SKOV3—day 5) was attributed to the embedded ovarian cancer cells at day 5, while the remaining shrinkage was due to the collagen shrinkage, as shown in control groups (day 0 vs. day 5). Collagen type I scaffolds exhibit contractility, while collagen type II was reported to mitigate or resist the contraction [34]. In this study, collagen type I hydrogel alone without collagen type II was used to form the organoids, which explains the contractility of the control from day 0 to 5.

### 3.3. Young’s modulus estimation

**Fig 10:**
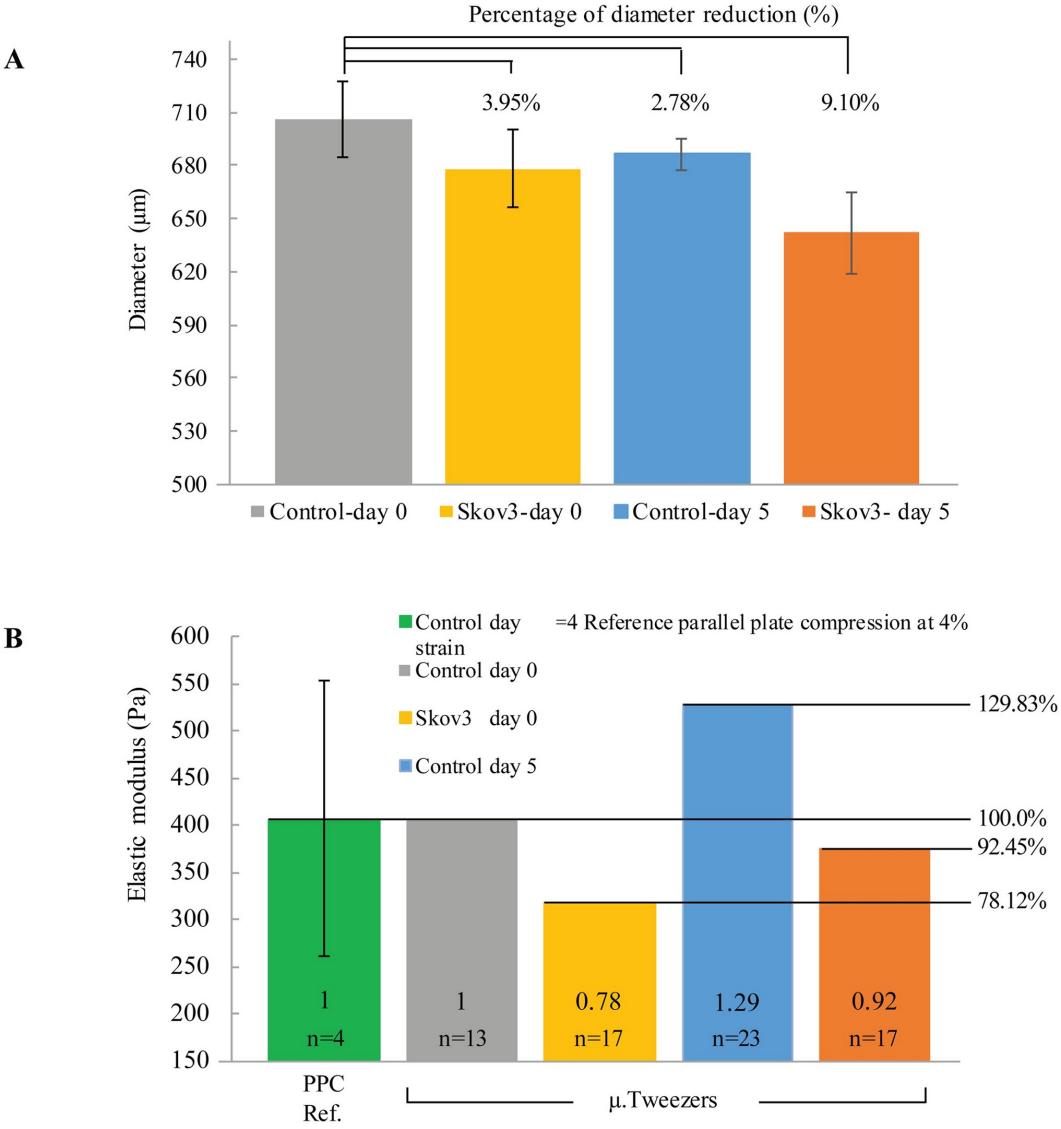
(A) The diameter changes across the four groups, while (B) is the elastic modulus calcualted based on the relative reference measurement conducted in typical parallel-plate compression (PPC) in combination with stiffness measurements.

The Young’s modulus of the control group (Control at day 0) was mechanically tested using typical PPC as described above. The elastic moduli of the other three groups were calculated relatively from the spring constant (Fig 10). According to the Hertz contact model of a cylinder and a parallel flat plate, the spring constant of the materials is not dependent on the curvature of the cylinder. The modulus of the group (SKOV3 at day 5) fell in the same range of (Control at day 0) based on the stiffness measurements. This trend is well observed in Fig 10B, where the collagen I organoid controls increased (29.83%) in the elastic moduli over five days; similarly, the ovarian cancer cells (SKOV3) embedded organoids increased (14.33%) in their elastic moduli. The trends observed also follow the reported literature where the cells change the elastic modulus of the surrounding matrix [35].

## 4. Discussion and conclusion

The efficacy of the μTweezers system was demonstrated through the ease of fabrication and use. The actuator components and fabrication parameters can be further tuned to accommodate other testing conditions and samples. The tuning of the manipulator and end-effectors through geometry change or curing conditions is easily achievable. Different sample structures and end-effector contact designs can be applied; higher strains were achievable with careful considerations of the cantilevers’ calibration linear range, spring constant, and the applicable contact models.

The samples used in this study were prepared in an identical cylindrical shape. One of the advantages of this approach is that we can assess relative stiffness by comparing the force curves. Our PDMS tweezers can also manipulate specimens with arbitrary shapes. Analytical contact mechanics models [36, 37] or finite element method (FEM) models [24, 27] can be used to evaluate the elastic modulus from the force curve. For spherical samples, a common approach is the use of contact mechanics models. Kosheleva *et al*. used the Hertz contact model to study tumor spheroids [38]. Irregularly shaped samples may be studied by an FEM model analysis. We have reported the use of a COMSOL^™^ model to estimate the elasticity of zebrafish embryos [27]. Hertz contact mechanics has been applied for a low strain regime (<5%) [39]. Many rheology-based hydrogel testing is reported in the linear viscoelastic regime [19]. In this study, we applied a 4% strain cut-off for all types of measurements and groups.

The system’s fine features were tested and validated through the experiments. The μTweezers system was capable of the stiffness characterization and manipulation of micro-meso scaled hydrogel-based organoids. We measured the stiffness changes of collagen I hydrogel organoids in four conditions. The microdevice fits on/in standard tissue culture Petri dishes, allowing high throughput mechanical compression characterization of arrayed 3D organoid pillars. On average, 40 tests were conducted per hour (20 organoids / 20 references), including alignment time between samples. This mechanical testing productivity is significantly increased compared to typically available devices (individual sample testing) while significantly reducing the biomaterials and technology costs. The device is highly tunable for different applications and operation ranges with maintained biocompatibility. Our group previously studied 3D tumor spheroids [24] and more irregularly-shaped zebrafish embryos [27] using other versions of micro tweezers, where we used FEM models to evaluate the stiffness. Although the actuator we used in this study is different and much improved from the previous designs, the end effectors were made of PDMS, which was used also in [27]. We can use the μTweezers to test various samples, including spheroids, tumoroids, and embryonic tissues. While the tweezer is capable of manipulating samples in the 50 μm-1500 μm range The measurement accuracy depends on the indentation depth ds (μm) needed for measurement and the manipulator repeatability (μm),. Since the image analysis showed the end effector’s positioning repeatability to be 2–3 μm (see Fig 2H), an indentation depth larger than ~10 μm is desirable. This indentation depth corresponds to a few hundred μm or larger when we apply 5% strain limit.

The platform has shown excellent potential for complementing established mechanical characterization methods such as AFM and nanoindentation while targeting efficient and widely adopted micro-meso scaled 3D biological samples/models. Hydrogel stiffnesses were successfully measured in a high throughput manner with substantial valuable biomaterials reduction, using a typical inverted optical microscope. The system can significantly contribute to the biomedical, biomechanical, biological, biomaterials, and regenerative engineering fields.
